# Unusual Case of Bilateral Tubercular Mastitis

**DOI:** 10.7759/cureus.1383

**Published:** 2017-06-22

**Authors:** Archit Gupta, Mudita Gupta, Jagdish Gupta

**Affiliations:** 1 General Surgery, Indira Gandhi Medical College, Shimla; 2 Dermatology, Indira Gandhi Medical College, Shimla

**Keywords:** tuberculosis, tb, breast, mastitis

## Abstract

Bilateral involvement of the breast with tuberculosis is extremely rare. It most commonly affects young lactating multiparous females, although rarely it may be reported in prepubescent males also. We present a case of a 27-year-old nulliparous female who presented with a history of multiple pus discharging sinuses around both areolae and was diagnosed as a case of bilateral tubercular mastitis. Tubercular mastitis being a paucibacillary disease, diagnosis is often difficult. Treatment consists of antitubercular therapy with or without surgery.​

## Introduction

Breast tuberculosis is a rare form of tuberculosis with an incidence of <0.1% in Western countries [1]. Even though the disease is more common in India as compared to the West, it is often misdiagnosed as carcinoma of the breast or breast abscess [2]. It most commonly affects young lactating multiparous females, although rarely it may be reported in prepubescent males also. Bilateral breast involvement is even more rare and very few cases have been reported till date [3]. We report a case of bilateral tubercular mastitis presenting in a young nulliparous female. We report this case due to its rarity and clinical significance.

## Case presentation

A 27-year-old nulliparous female presented to our outpatient department with a history of pus discharge from multiple sites around the nipple-areola complex of both the breasts for four years. The patient had undergone incision and drainage twice, with a suspicion of breast abscess at a peripheral hospital. There was no history of any palpable lump in the breasts, fever, night sweats, weight loss, or any respiratory symptoms. There was no family history of breast cancer or tuberculosis. There was no history of diabetes, immunosuppression, previous treatment for tuberculosis or history of contact with a known case of tuberculosis.

**Figure 1:**
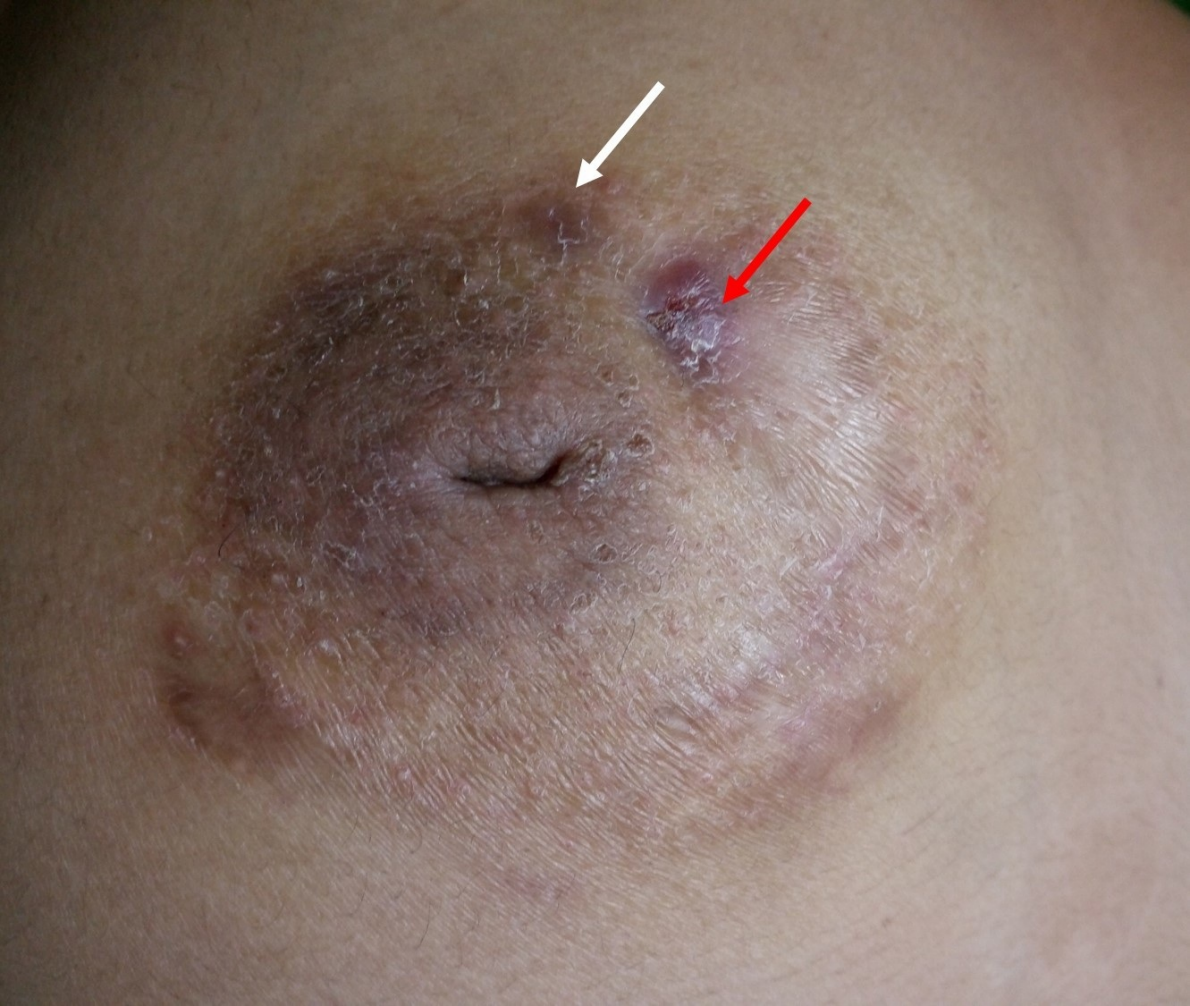
Right breast of the patient showing periareolar plaques (white arrow) and sinus (red arrow)

**Figure 2:**
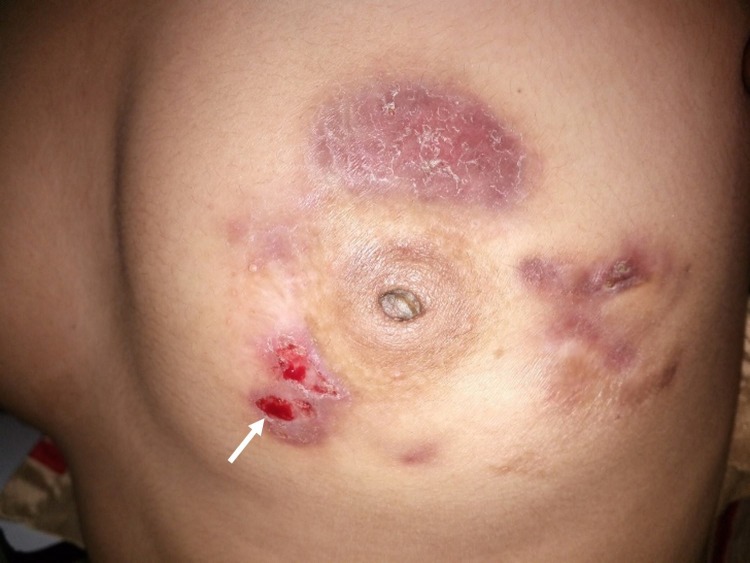
Left breast of the patient showing erythematous plaque (white arrow)

The general physical examination and systemic examination were within normal limits. On local examination, nipple retraction with multiple scars, indurated granulomatous erythematous plaques, and discharging sinuses around the nipple-areola complex were found in both the breasts (Figures 1-2).

There was no palpable lump, and the bilateral axillae were normal. A diagnosis of tubercular mastitis was suspected. The differential diagnosis included carcinoma of the breast, breast abscess, and granulomatous mastitis with etiology other than tuberculosis.

**Figure 3:**
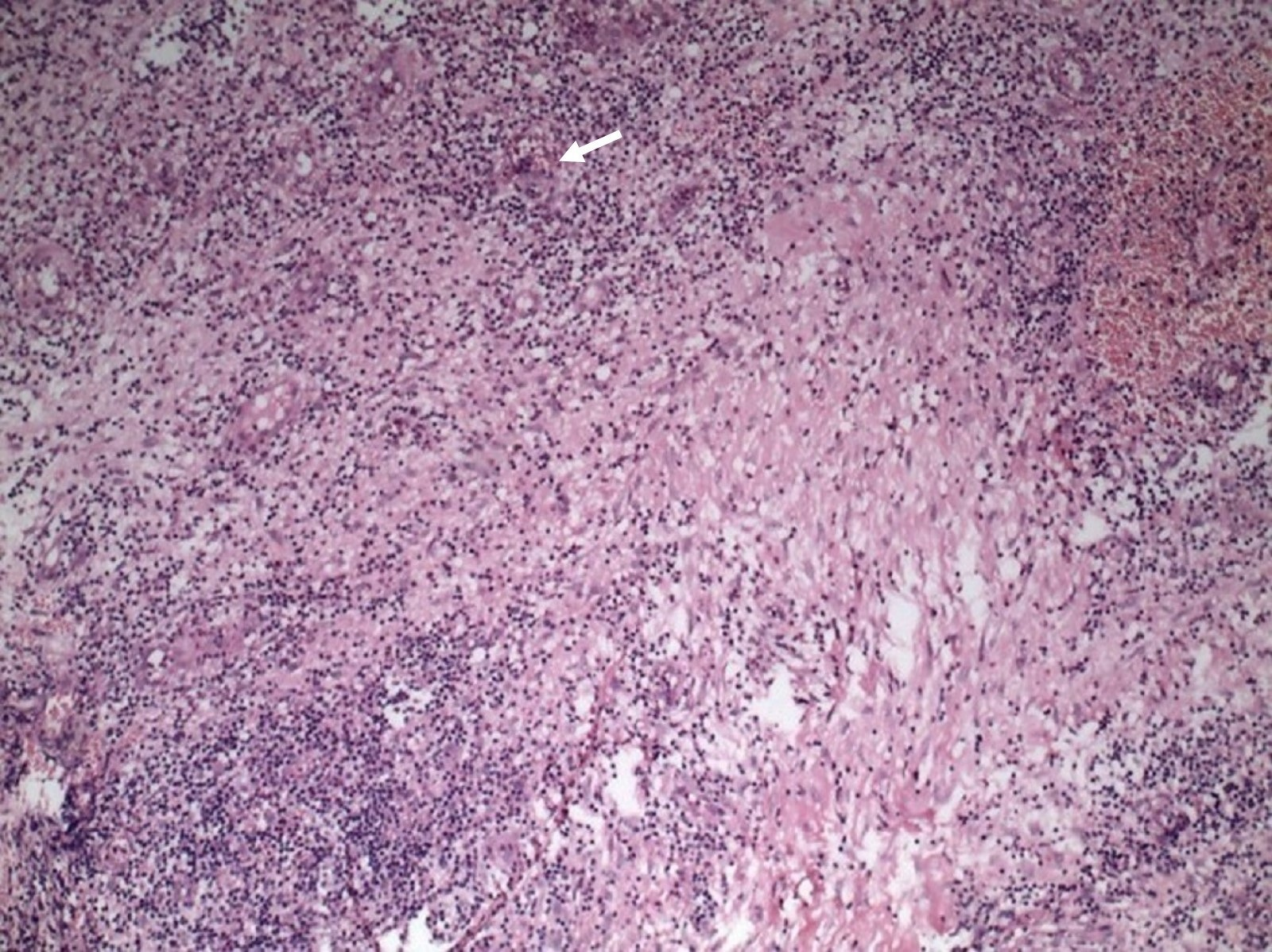
Histopathology (40x) showing epitheloid cell granulomas (white arrow)

All routine hematological and biochemical investigations were within normal limits, except the erythrocyte sedimentation rate, which was 25 mm in the first hour. A chest X-ray did not reveal any abnormality. Ziehl-Neelsen staining of the pus had no acid-fast bacilli and the culture was sterile. Real time polymerase chain reaction (PCR) for Mycobacterium tuberculosis and other mycobacteria was negative. An ultrasound of the breast showed bilateral sinus tract with duct ectasia with collection in the subcutaneous plane. Fine-needle aspiration cytology (FNAC) revealed multiple epitheloid cell granulomas with occasional giant cells suggestive of granulomatous mastitis. The skin biopsy had granuloma consisting of lymphocytes, plasma cells, epitheloid cells and occasional Langhans and foreign body giant cells suggestive of suppurative granulomatous dermatitis consistent with tuberculous mastitis (Figure 3).

**Figure 4:**
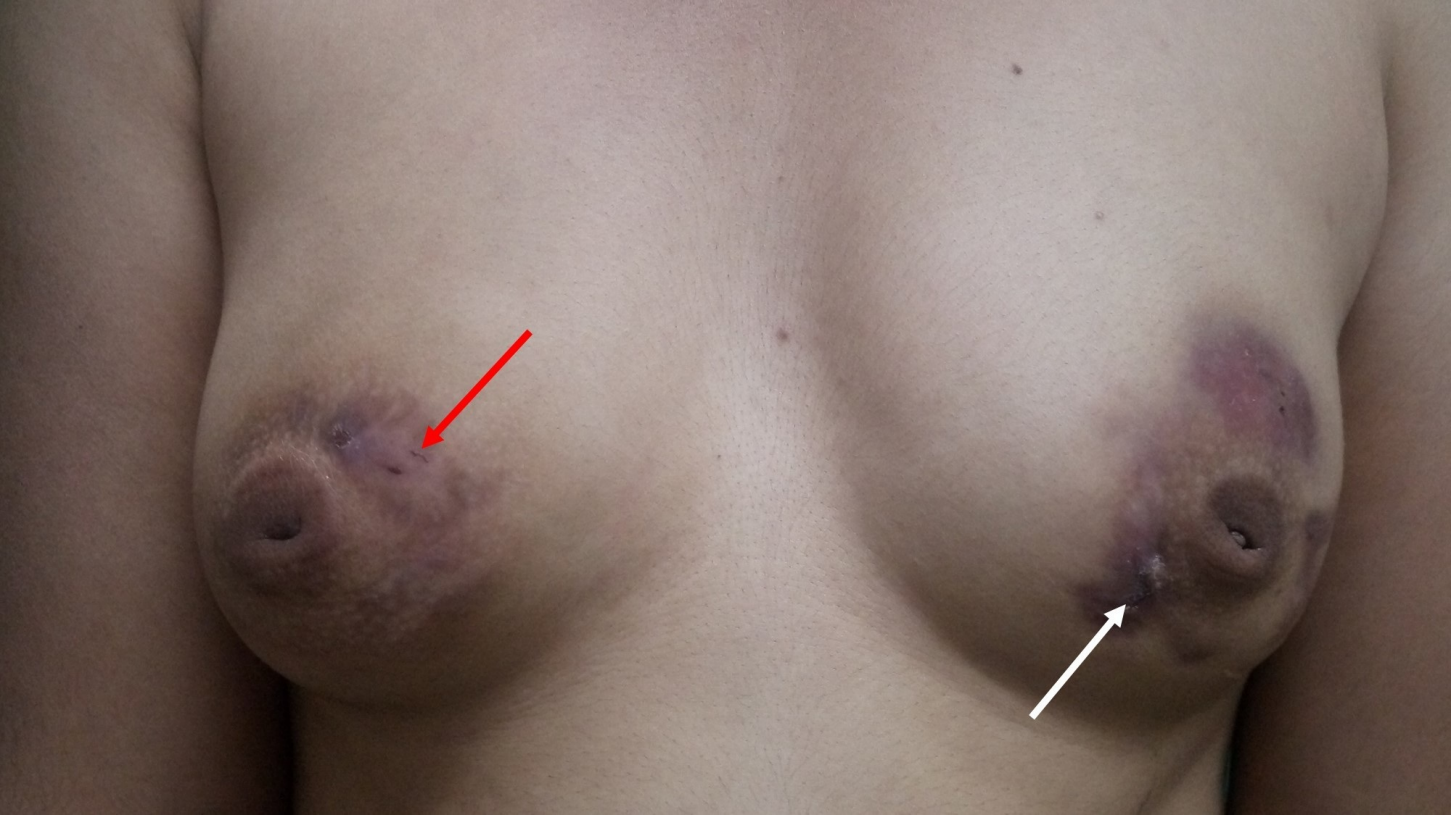
Bilateral breasts showing disappearance of erythematous plaque (white arrow) and sinus (red arrow) after treatment with antitubercular therapy for one month

The patient was started on antitubercular therapy including isoniazid, rifampicin, pyrazinamide and ethambutol with doses adjusted according to the weight of the patient for two months. On the first follow-up after a month, the lesions became less indurated, erythema decreased, and the pus discharge reduced (Figure 4).

The patient had satisfactory response to antitubercular treatment and was called for follow-up and readjustment of drugs after a month.

## Discussion

Breast tuberculosis was first recorded by Sir Astley Cooper in 1829, who called it ‘scrofulous swelling of the bosom’. Breast tuberculosis is rare because of the high resistance offered by the breast tissue to the survival and multiplication of tubercle bacilli [4]. It commonly affects multiparous females usually in the age group of 21–40 years [5]. Our patient was a 27-year-old nulliparous female.

Breast tuberculosis can be primary or secondary. Primary is when the tuberculous infection is confined to the breast and is extremely uncommon. It is secondary when there is a coexisting focus of tuberculosis somewhere else in the body, most commonly, pulmonary tuberculosis. Our patient was a case of primary tuberculosis of the breast [5].

Diagnosis of breast tuberculosis is a challenge and the patient is usually subjected to numerous investigations before a diagnosis is made. Mantoux test is usually positive in endemic areas and stands obsolete. Mammogram is rarely used for diagnosis as it usually does not distinguish from carcinoma of the breast [6]. Ultrasonography is cheap, easily accessible, and helps in characterizing the lesion better without exposure to radiation [7]. Diagnosis is ideally by bacteriological confirmation from breast tissue by Ziehl-Neelsen stain or culture. But as the disease is usually paucibacillary, bacteria are isolated only in 25% of the cases [8].

Fine needle aspiration cytology (FNAC) is an important tool for diagnosis of breast tuberculosis. Approximately 73% of the cases can be diagnosed by demonstrating epitheloid cells with caseous necrosis on FNAC [8]. In our case, FNAC showed granulomas with epitheloid cells; however, there was no necrosis, which can be explained because only a small amount of tissue was harvested and examined in FNAC. Histopathological findings usually include epitheloid cell granulomas, but this picture can also be present in other diseases which include sarcoidosis, other fungal infections, and idiopathic granulomatous mastitis [7-8].

The treatment primarily comprises of antitubercular therapy with or without surgery. Residual lump following surgery may require surgical removal. A simple mastectomy can also be done.

Our patient had granulomas consisting of epitheloid cells in FNAC and biopsy and responded well to treatment by antitubercular therapy. Hence, a final diagnosis of tubercular mastitis was made after one month of treatment.

## Conclusions

​Breast tuberculosis is a rare form of tuberculosis that is often misdiagnosed as carcinoma of the breast or breast abscess. Bilateral breast involvement is even more rare and very few cases have been reported till date. A high index of clinical suspicion is required to make the diagnosis. We report a case of bilateral breast tuberculosis where diagnosis of tuberculosis was confirmed only after the patient showed response to antitubercular treatment.

## References

[1] Kalac N, Ozkan B, Bayiz H (2002). Breast tuberculosis. Breast.

[2] Green RM, Ormerod LP (2000). Mammary tuberculosis: rare but still present in the United Kingdom. Int J Tuberc.

[3] Sreeramulu PN, Venkatachalapathy TS, Prathima S (2012). A case report of bilateral tuberculosis of breast. J Clin Case Rep.

[4] Khanna R, Prasanna GV, Gupta P (2002). Mammary tuberculosis: report on 52 cases. Postgrad Med J.

[5] Shinde SR, Chandawarkar RY, Deshmukh SP (1995). Tuberculosis of the breast masquerading as carcinoma: a study of 100 patients. World J Surg.

[6] Tewari M, Shukla HS (2005). Breast tuberculosis: diagnosis, clinical features & management. Indian J Med Res.

[7] Popli MB (1999). Pictorial essay: tuberculosis of the breast. Indian J Radiol Imag.

[8] Kakkar S, Kapila K, Singh MK (2000). Tuberculosis of the breast. A cytomorphologic study. Acta Cytol.

